# Ethnopharmacological knowledge and physicochemical properties of hot springs in Bhutan

**DOI:** 10.1186/s12906-025-05123-2

**Published:** 2025-10-21

**Authors:** Tulsi Gurung, Tshering Yangden

**Affiliations:** 1 Department of Agriculture, College of Natural Resources, Royal University of Bhutan, Lobesa, Punakha, Bhutan; 2 Tarayana Centre for Research and Development, Tarayana Foundation, Thimphu, Bhutan

**Keywords:** Balneotherapy, Ethnopharmacological, Physicochemical, Therapeutic, Thermal springs

## Abstract

**Background:**

Balneotherapy, the therapeutic use of mineral and thermal waters, is an ancient practice with global significance. In Bhutan, the use of hot springs is deeply ingrained in culture and traditions as balneotherapy practices, enriched by extensive ethnopharmacological knowledge. Therefore, this study aims to document the socio-cultural and ethnopharmacological importance of Bhutanese hot springs, analyse their physicochemical characteristics, and correlate them with the perceived therapeutic benefits.

**Methods:**

The study focused on three hot springs in the Gasa and Punakha districts. Qualitative data collection on ethnopharmacological knowledge included informal conversations, semi-structured interviews, oral narrations, and focus group discussions. Thematic analysis was applied to interpret the data. Physicochemical properties of the thermal waters were analysed following standard laboratory protocols. Piper and Stiff plots were used to determine the water type and ion concentration, respectively. Moreover, Spearman’s correlation coefficient was used to assess the physicochemical relationships of the thermal water to the perceived health benefits.

**Results:**

The study found that people visit hot springs for spiritual connection, social bonding, and therapeutic benefits for musculoskeletal, dermatological, gastrointestinal, respiratory and cardiovascular conditions. The Piper diagram revealed that all hot springs exhibit a sodium-chloride (Na⁺–Cl⁻) hydro-chemical facies, suggesting a common geochemical origin. Stiff plot showed higher concentrations of ions in Gasa hot spring than other two sites. A moderate positive correlation was found between the concentrations of calcium, sodium, potassium, and magnesium, and the perceived health benefits for musculoskeletal and dermal conditions.

**Conclusion:**

This study highlights the cultural, spiritual, and therapeutic importance of hot springs in Bhutan. The ethnopharmacological benefits, particularly for musculoskeletal and dermal conditions, is found to be linked to the physicochemical composition of the thermal waters. However, it calls for more research in terms of physicochemical analysis of thermal waters and clinical trials to scientifically validate these practices so they can be institutionalized as complementary and alternative treatments.

## Introduction

Balneotherapy, the therapeutic use of mineral-rich thermal waters has gained scientific recognition globally for its effectiveness in treating musculoskeletal, dermatological, and psychosomatic conditions [1, 2]. While clinical trials in Europe and Japan have highlighted physiological benefits such as reduced inflammation, improved mobility, and stress alleviation [3] there is limited research from Himalayan contexts, including Bhutan.

In Bhutan, hot springs (*Tshachu*) are culturally and spiritually significant and are often referred to as “*therapeutic springs*”, often associated with Buddhist beliefs of possessing healing properties. This perception is rooted primarily in local tradition and cultural belief systems, where the waters are considered spiritually potent and curative for various ailments, especially musculoskeletal pain and skin diseases. These claims are often transmitted through oral histories and communal practices, and many springs are attributed to sacred origins, such as blessings from Guru Rinpoche [4]. Thus, hot springs are frequented not only as a routine wellness practice but also for healing purposes. However, from a scientific perspective, the term “*therapeutic*” typically refers to mineral or thermal waters whose physicochemical properties meet specific standards that have demonstrated efficacy in clinical or biomedical contexts [1, 2, 5]. Thus, in this study, the use of the term “therapeutic springs” reflects primarily local perceptions and traditional beliefs.

These hot springs serve dual purposes of therapeutic relief for ailments such as joint pain and spiritual purification through ritual offerings to protective deities. Such practices reflect a holistic healing tradition that merges physical, spiritual, and communal dimensions, and this dimension adds a psychosocial and spiritual element not widely addressed in the mainstream literature but increasingly acknowledged in integrative health frameworks [6].

Despite growing interest in balneological tourism and wellness practices, Bhutan’s thermal springs remain under-researched from both biomedical and ethnographic perspectives. There is a critical need for integrated studies that assess not only the physicochemical properties of the waters to set the standards but also the socio-cultural and ecological contexts in which they are used. Doing so would align Bhutan’s traditional health practices with global scientific discourse, while supporting the sustainable and culturally respectful development of these heritage sites.

The therapeutic use of natural mineral waters has gained renewed global recognition for its efficacy in treating musculoskeletal and inflammatory conditions [2, 3]. Recent biomedical literature supports the long-held Bhutanese belief in the healing properties of thermal waters. Studies have shown that balneotherapy can modulate immune responses, alleviate chronic pain, and improve quality of life, particularly in cases of rheumatologic and musculoskeletal disorders [7, 8]. These outcomes are mirrored in Bhutanese practices where hot springs are commonly visited for the relief of joint pain, skin diseases, and general rejuvenation.

Furthermore, the integration of balneotherapy into modern rehabilitation protocols, particularly for chronic pain and stress-related conditions, aligns with Bhutan’s traditional use of thermal baths for both physical and mental wellness [9]. There is limited study done on hot springs in Bhutan which integrates both social and natural science perspectives. The term “therapeutic” in the Bhutanese context is largely grounded in oral tradition and user perception, rather than systematic chemical analysis of water or clinical trial. Therefore, the objectives of this research are to (i) document socio-cultural and ethnopharmacological significance and (ii) analyse the physicochemical properties of thermal water and correlate with the perceived therapeutic benefits.

## Methods

### Study sites

**Fig. 1:**
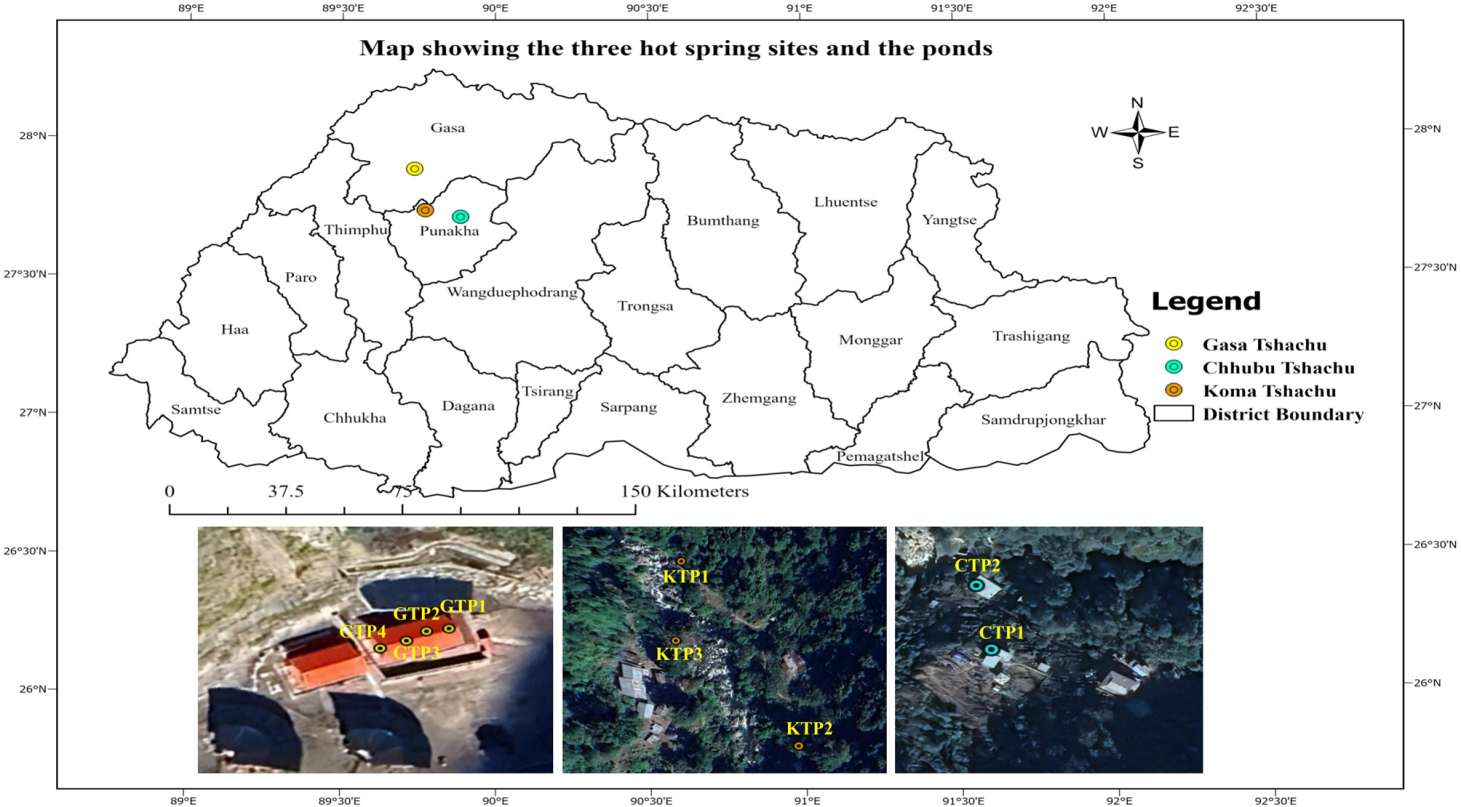
Map showing the three hot spring sites and the ponds

Gasa hot springs from Gasa district, and Chubu and Koma from Punakha district of North-Western Bhutan were the focus of our study. These springs are well-known for their therapeutic benefits and attract visitors from across the country. The annual visitors recorded for Gasa from July 2024 to June 2025 were 5606, excluding the day visitors, which shows the significance of the hot springs. Reliable records were not available from Chubu and Koma. Winter months, starting December–February, are the most visited months. The data collection was carried out in September 2023 and February 2024, and again reconfirmed for some elements in water in April 2025. Since users reported different perceived benefits across various ponds, a total of nine hot spring ponds were selected for the study: four from Gasa (designated for female use), three from Koma, and two from Chubu. Detailed information regarding the location of these hot springs and ponds is presented in Table 1; Fig. 1.

**Table 1:** Description of hot spring and pond location in each site

Name of hot spring	Location (Block, District)	Latitude^o^*N*	Longitude^o^ E	Altitude (masl)
Gasa (GTP1)	Khatoed, Gasa	27.88767	89.73628	2142
Gasa (GTP2)	Khatoed, Gasa	27.88765	89.73625	2142
Gasa (GTP3)	Khatoed, Gasa	27.88762	89.73623	2142
Gasa (GTP4)	Khatoed, Gasa	27.88759	89.73620	2142
Koma (KTP2)	Goenshari, Punakha	27.73919	89.77096	1732
Koma (KTP3)	Goenshari, Punakha	27.73857	89.77073	1761
Koma (KTP1)	Goenshari, Punakha	27.73748	89.77137	1731
Chubu (CTP1)	Toedwang, Punakha	27.71405	89.88630	1817
Chubu (CTP2)	Toedwang, Punakha,	27.71445	89.88625	1797

‘*P*’ here denotes ponds in the hot spring area (four ponds from female side in Gasa Tshachu/hotspring, three ponds from Koma Tshachu and two ponds from Chubu Tshachu

### Data collection on socio-cultural and ethnopharmacological aspects of hot springs

A combination of qualitative data collection tools was employed to gather in-depth insights into the cultural, social, and therapeutic significance of hot springs in Bhutan. The primary tools included informal conversations, semi-structured interviews, focus group discussions, and oral narrations, each selected to align with the naturalistic and participatory nature of the study settings.

### Ethical approval

Ethical considerations were prioritized throughout the study. The formal ethical clearance was obtained from the Ethics Committee of the College of Natural Resources, Royal University of Bhutan. Verbal informed consent was obtained from all participants before their involvement in interviews, focus group discussions, or observations. Verbal consent was obtained due to low literacy levels among many participants. Respondents were comfortable providing verbal consent then signing the documents they do not fully understand. Therefore, verbal consent honoured their preferences while also fulfilling ethical requirements for informed consent.

### Sampling and sample size

A purposive sampling method was used to explore ethnopharmacological knowledge and perceptions of therapeutic benefits associated with selected hot springs. A well-defined inclusion and exclusion criteria were used to guide participant selection.

The participants included were 18 years or older and were regular or long-term users of the hot springs, many of whom visited for health-related reasons. They also held beliefs in spiritual or cultural healing, in addition to physical healing, and shared their personal experiences, motivations, and beliefs about the curative properties of the water. Additionally, local community members and elders were included to gather insights on community beliefs, practices, and oral histories. Those who were actively using the springs for perceived and diagnosed health conditions at the time of the study were also included. Moreover, caretakers and managers were included for information regarding the number of visitors, types of visitors, purpose of the visit, duration of the visit, and the facilities available at the sites.

In contrast individuals who were visiting the hot springs for the first time or those who lacked clarity about the purpose or benefits of the springs were excluded.

Based on these criteria, a total of 36 participants were selected for face-to-face interviews. The sample included manager/caretakers (3) and users (33). Participants ranged in age from 18 to 80 years and included both men (19) and women (14).

### Data collection process

Informal conversations were conducted with site managers at each of the three hot spring locations. These engagements occurred organically during site visits and were instrumental in eliciting nuanced perspectives on the day-to-day operation of the springs, customary practices, and observed patterns in visitor behaviors. The informal format facilitated open communication and built rapport, especially in culturally sensitive or logistically constrained settings.

To explore user experiences and health-related beliefs, a semi-structured questionnaire was administered to participants at the bathing sites. The questionnaire was designed to elicit perceptions regarding the ethnopharmacological knowledge on hot springs to treating health conditions. In addition to health outcomes, the instrument also probed the motivations for engaging in these long-standing balneotherapy traditions, the symbolic and ritual aspects of participation, and the broader social functions of communal bathing. This approach allowed for both consistency across interviews and flexibility for participants to elaborate on culturally embedded knowledge and personal experiences. Interviews lasted between 15 and 30 min.

In Gasa hot springs, ponds are designated separately for males and females; therefore, group discussions for males and females were conducted separately as they remained immersed in the hot water. For Koma and Chubu Tshachu, ponds are not separated; therefore, discussions took place together. Data were collected on the ethnopharmacological properties, practices, socio-cultural values, and beliefs.

Furthermore, an oral historical narration was collected from an elderly resident of Gasa, recognized by the local community as a knowledgeable custodian of oral traditions. This narration provided valuable contextual information on the origin, cultural heritage, and evolving significance of the Gasa hot springs. Such oral accounts complemented the empirical data by embedding participant perspectives within a broader historical and cultural framework.

All interviews and group discussions were audio recorded with informed consent and subsequently transcribed verbatim for analysis.

### Ensuring credibility

Credibility in analysing health claims was maintained by including only those therapeutic effects that were consistently reported by multiple respondents. Claims mentioned by three or fewer individuals were excluded to minimize anecdotal bias.

### Data analysis

A thematic analysis approach was employed to interpret the qualitative data collected from interviews, focus group discussions, and oral narrations. This method was chosen for its flexibility in identifying, analyzing, and reporting patterns within the data, and its suitability for exploring culturally grounded knowledge systems and subjective health experiences.

The analysis followed a systematic, iterative process. First, audio recordings of the interviews and discussions, which were originally conducted in Dzongkha, were transcribed verbatim and subsequently translated into English. Transcripts were then carefully read and re-read by the research team to ensure familiarization with the content. During this process, initial codes were manually generated to capture salient ideas, recurring concepts, and culturally significant expressions relevant to the study’s objectives. These codes were subsequently reviewed and grouped into broader thematic categories that reflected the core aspects of the research, such as beliefs about healing properties, reasons for continued use of traditional baths, and the sociocultural dimensions of communal bathing. Emerging themes were refined through team discussions to ensure consistency and reliability in interpretation.

### Data collection on physicochemical properties

Water samples were taken from four designated ponds for females from Gasa hot springs, two from Chubu, and three from Koma hot spring. Before sampling, one-liter polyethylene terephthalate (PET) bottles were thoroughly cleaned with distilled water and subsequently rinsed with the sample water from the sample site, and then samples taken. To ensure proper preservation, the samples were acidified to a pH of less than 2.0 by incorporating 0.15% vol./vol. of concentrated nitric acid.

### Methods for physiochemical analysis

Nineteen quality parameters were assessed. The physical parameters, including temperature, pH, electrical conductivity (EC), and total dissolved solids (TDS), were measured at the site using the multiparameter PCSTestr 35, while dissolved oxygen levels were determined with the Edge @ Hanna’s DO meter. Turbidity was quantified using a microprocessor turbidity meter, and total hardness (expressed as CaCO3) was evaluated through EDTA titrimetric methods at the College of Natural Resources laboratory in Bhutan.

The remaining nine chemical parameters were analyzed at the ENVIROCHECK laboratory in Kolkata, India, which holds accreditation from the National Accreditation Board for Testing and Calibration Laboratories (NABL) under ISO/IEC 17025:2017. Calcium (Ca^2+^), magnesium (Mg^2+^), and chloride (Cl^−^) concentrations were determined using the EDTA titrimetric method (APHA 3125, 2017). Additionally, sodium (Na^+^), potassium (K^+^), copper (Cu^2+^), zinc (Zn^2+^), iron (Fe^2+^), and manganese (Mn^2+^) were measured using the Agilent 7800-ICPMS, employing microwave-assisted digestion (3030). Ammonia (NH_3_^−^) was analyzed with the Agilent Cary 60 (UV-VIS) spectrophotometer (APHA 4500 NH3-B) and Sulphur (as S^2^) using 4500-S^2^-DMethylene Blue Method. Sulphate (SO₄²⁻) was anlaysed using turbidimetric method (APHA 4500 SO₄²⁻ E), Nitrate (as NO_3_^−^) following 4500-NO3 E APHA method. Bicarbonate (HCO_3_^−^) was analysed using APHA 2320 B, alkalinity (bicarbonate) by titration method. Sulphate, carbonate, bicarbonate, chloride were analysed in Royal Centre for Disease Control in Bhutan. All chemicals utilized in these analyses were of analytical grade.

### Data analysis

The physicochemical properties of water were compared with the World Health Organisation (WHO) standards for drinking and European Spa Association (ESPA guidelines). Water chemistry were interpreted using graphical techniques designed to visually represent chemical components. All chemical concentrations were analysed in milligrams per liter (mg/L) and converted to milliequivalents per liter (meq/L) wherever necessary. The percentage of each cation and anion was calculated relative to the total milliequivalents of cations and anions, respectively. This normalization allowed for comparative visualization of hydrochemical facies. A Piper diagram consisting of two equilateral triangles representing the relative concentrations of major cations and anions and a central diamond-shaped field that reflects the overall water type was used using Golden Software Grapher Beta. Moreover, Stiff diagrams were also used to compare the relative ionic composition of individual ponds of the hot springs. The healing properties were categorized into five, ranked as per the responses, and Spearmen correlation was used to analyse the association between the perceived health benefits with the physicochemical properties of water.

## Results

### Thermal healing: cultural and physical dimensions of hot spring use in Bhutan

The discussions indicated that soaking in hot springs presents numerous benefits for both physical health and mental wellness, thereby enhancing the overall quality of life. In Bhutan, where Buddhism is deeply ingrained in the cultural fabric, hot springs are thought to have been miraculously created by Guru Rinpoche, also referred to as the second Buddha, to offer healing properties for various health issues. Consequently, these springs are venerated as blessings from the Buddha. They function not only as venues for physical recovery but also as spiritual havens, allowing individuals to deepen their connection with their faith and the natural environment. Hot springs are frequently linked to deities and spiritual beliefs. There exists a belief in protective deities associated with these springs, which are often regarded as sacred locations. These deities are thought to guard the hot springs and their surroundings, preserving their sanctity and bestowing blessings upon visitors. Rituals and offerings are performed to honor these deities, ensuring their ongoing protection and favor. Such practices not only reinforce faith in the therapeutic properties of the hot springs but also impart a sense of spiritual fulfillment to the community. A 79-year-old resident of Gasa shared insights regarding the three protective deities of the Gasa hot springs.

According to him:

*“In this valley*,* there are sacred places known as Neka where we go to propitiate the local protecting deities. There were three protecting deities and in the seventieth century when Zhabdrung Rinpoche arrived from Tibet*,* the main deity of the valley known as Gombo asked the three protecting deities to go with him and welcome Zhabdrung. However*,* one deity known as Koentop refused and hid in a cave. Gombo then asked the other two deities not to disturb Koentop if he was not willing to accompany them. Thus*,* the other two deities known as Tshegyen Drangyen and Toli Phaka accompanied Gombu to receive Zhabdrung to Gasa. Zhabdrung later changed the name of Tshegyen Drangyen to Tashi Thobar and Toli Phaka to Dhendup Norza and as a sign of appreciation*,* they were made entitled to propitiation and offerings which is still followed today. No prayer was composed for Koentop and he is neither propitiated nor considered important”.*

The interview participant conveyed that it is widely held that the hot springs were established by Guru Rinpoche at the request of the local populace to address a variety of health issues. Furthermore, Guru Rinpoche is attributed with the creation of numerous medicinal plants. There have been concerns among the community regarding the sustainability of both the hot springs and the medicinal flora. In response, Guru Rinpoche asserted that those who possess profound faith will always find access to these healing resources. The spiritual significance of this belief is underscored by how the elder admonishes the youth for their lack of respect towards the advantages offered by the hot springs.

*“Many people complain about the ineffectiveness of the hot springs and this is usually true with the younger generations. These young people do not have faith in the first place and they usually come for entertainment. They also do not know the procedure of soaking in the hot springs as they sit at the rim soaking half body or only their legs*,* they become loud disturbing other soakers*,* and then have the audacity to talk about the elderly and their blind faith. How is one going to get healed without faith and discipline? Before entering the pool*,* one must seek blessings from the protecting deities to heal ailments. One must then offer drops of water to the spirits to show respect and then soak neck-deep for at least an hour at a time and maintain the tranquility of the place”.*

On the other hand, Chubu Tshachu, situated in Punakha, is a well-known hot spring that has two ponds. The first pond is reputed to possess therapeutic properties that aid in treating dermal diseases, tuberculosis, and stomach ailments. The second pond, located below the first pond, is believed to be effective in treating muscle sprains and diabetes.

The Koma hot springs also located in Punakha are believed to be effective for healing fractures, ulcers, stomach pain, and urinary tract infections. Due to its comparatively lower temperature, patrons have indicated that they can soak for extended periods, thereby experiencing significant therapeutic benefits.

### Thermal springs as a social and familial space

Organizing a trip to the thermal springs serves as a popular activity for family members who have been separated due to their careers. Such reunions can strengthen relationships and foster deeper bonds. Collaborating on travel arrangements, making collective choices, and sharing the excitement of upcoming adventures can enhance interpersonal connections and create lasting memories that contribute to overall well-being and mental health. Creating unforgettable experiences with family fosters a sense of belonging and alleviates feelings of loneliness, as illustrated by the story of an elderly woman in her eighties.

*“Planning a soaking in hot springs is a good way to have family gatherings. Some of my siblings are married to people from outside our district. Children work in different places. Except for yearly religious rituals*,* it is very difficult to come together. So*,* we try to plan trips to different hot springs in a year and it is fun because we share responsibilities as to who should buy meat*,* and eggs*,* provide transport*,* and so on. It is also an opportunity for cousins to get to know each other. We also make new friends and sometimes the new friends become more like siblings sharing food*,* and exchanging phone numbers to keep connected. It saves boredom at the pools as we do not know how time passes while soaking because of the interesting conversations that take place in the pools. If we plan to soak for one hour*,* then we end up soaking for two hours or more”.*

### Immersion duration

The therapeutic properties of these hot springs appear to significantly enhance muscle relaxation and reduce tension, ultimately fostering mental tranquility. Participants reported feeling a sense of calm and serenity after each soak. On average, individuals dedicate around seven hours daily to soaking in hot springs, typically allocating 2–3 h in the morning, 1–2 h in the evening, and an additional 1–2 h post-dinner. To maintain their energy levels, they consume food and beverages during these intervals, as prolonged soaking can be physically draining. However, the effects of the hot springs may differ based on an individual’s capacity to adapt to the thermal environment. An 81-year-old woman visitor from Haa at Gasa *tshachu* shared her experience:

*“For instance*,* Pool One*,* which has the lowest temperature*,* seems to be well-suited to my body. After soaking in this pool*,* I experience a sense of relaxation. In contrast*,* the other three pools*,* which are significantly hotter*,* leave me with discomfort and a severe headache. Consequently*,* I have resolved to continue using Pool One”.*

Conversely, another 64-year-old woman visitor from Mongar district reported a different experience:

*“After soaking in Pool One*,* I developed rashes. The subsequent pool*,* Pool B*,* which is warmer*,* proved to be more beneficial for me. I found that I could sleep soundly at night*,* as the pain in my joints appeared to diminish”.*

This observation indicates that the hot springs not only offer pain relief but also enhance sleep quality and overall well-being, contingent upon individual physiological responses. The majority of respondents reported that the Gasa hot spring possesses effective healing properties for conditions such as arthritis, rheumatism, indigestion, and tuberculosis. Chubu hot spring is reported to have similar benefits, with the added advantage of reducing swelling and alleviating back pain.

There is a safety concern regarding prolonged soaking, as it may pose possible adverse effects, such as dehydration, dizziness, or heat exhaustion. Nevertheless, persuading individuals regarding these risks proves challenging due to a blend of cultural beliefs, personal experiences, and deficiencies in risk awareness. Individuals often place greater trust in ancestral knowledge than in contemporary medical advisories, viewing hot springs as sacred, therapeutic, and historically validated. Additionally, social norms and peer influence contribute to prolonged soaking, as elders may advocate for extended durations as a demonstration of resilience, making it difficult to deviate from the conventional inquiry of why one cannot partake in lengthy soaks if everyone else is doing so.

The ethnopharmacological characteristics (Table 2) reported by users exhibited similarities across various ponds at different locations, although certain ponds were noted for their unique therapeutic properties. For instance, some people frequent one of the three pools at Koma hot springs, based on the belief that it alleviates fractures and joint discomfort. One individual with a fractured leg recounted being transported from the road to the pond site. Remarkably, within a week, her condition improved significantly, allowing her to walk independently, albeit slowly. She had been soaking in Pond 2, which maintains a warm temperature of 38 °C. On another visit, there were four visitors with fractured leg out of 12 visitors. Therefore, there is a strong belief and lived experience of faster wound healing in Koma.

**Table 2:** Ethnopharmacological properties of hot springs

Hot springs and ponds	Ethnopharmacological properties
Gasa (GTP1)	All pools in Gasa are said to have same healing properties such as arthritis, rheumatism, reduces swelling in joints, skin diseases and itching disorders, pustules on face and body, indigestion, ulcers, urinary tract infection, tuberculosis (TB), also improves facial beauty
Gasa (GTP2)	
Gasa (GTP3)	
Gasa (GTP4)	
Koma (KTP1)	Arthritis, skin diseases, gastritis and ulcers
Koma (KTP2)	Swelling, fracture, gastritis and ulcers, urinary tract infection
Koma (KTP3)	Arthritis, skin diseases, gastritis and ulcers
Chubu (CTP1)	Treatment of dermal diseases, stomach ailments, swelling and TB, urinary tract infection
Chubu (CTP2)	Arthritis, back pain, muscle sprains, diabetes, bronchitis and asthma, sinusitis

### Physicochemical properties

The physical and chemical properties of the hot spring water are presented in Table 3.

**Table 3:** Physicochemical properties of water from three hot springs (nine ponds)

Parameter	Gasa hot spring				Koma hot spring			Chubu hot spring		WHO	ESPA
	P1	P2	P3	P4	P1	P2	P3	P1	P2		
Water Temp (^o^C)	41.4	42.6	42.7	43.1	32.2	38.1	39.8	45.3	46.4	-	> 25 °C
pH	7.9	8	8	7.9	10.4	10.3	10.3	9.8	9.8	6.5–8.5	
EC (µs/cm)	11.2	11.4	11.5	11.2	419	474	520	817	816	2500	-
TDS (mg/L)	7.28	7.41	7.475	7.28	272.4	308.1	338	531.1	530.4	1000	> 2500
DO (mg/L)	4.7	4.6	4.6	4.6	6.3	7.4	5.7	0.2	1.2	-	-
Turbidity (NTU)	10.9	8	7.9	8.5	0	1.2	0	0	0	0.5	-
TH (mg/L) CaCO_3_	14.7	14	16	14	33.3	28	11.3	40.9	42.1	150–500	-
Na^+^ (mg/L)	1904.2	1695.4	1722	1937.8	180	234.7	307	832.6	924.8	200	-
K^+^ (mg/L)	294.1	199.3	269.1	250.2	0.7	0.7	0.7	2.7	2	50	-
Ca^+2^ (mg/L)	21.3	18.7	20.3	19.2	2	ND	1.3	32.9	35.5	100	500
Mg^+2^ (mg/L)	6.7	4.7	7	5.5	31.3	ND	10	8	6.7	50	150
SO4^−^ (mg/L)	5	4	2	2	3	3	16	23	24	< 250	1200
Cl^_^ (mg/l)	1700	1752	1813	1840	33.5	29.9	30.9	65.6	68	250	
CO3^−^ (mg/L)	0	0	0	0	6.7	7.6	7	0	0	-	-
HCO3^−^(mg/L)	280.2	284	330.7	317.4	3.2	3.2	1.6	9.9	16.5	90.2	1300
NO3^−^ (mg/L)	1.1	1.1	0.9	1.1	2.6	0.9	0.1	0.2	0.1	50	-
Cu^+2^ (mg/L)	< 0.04	< 0.04	< 0.04	< 0.04	< 0.04	< 0.04	< 0.04	< 0.04	< 0.04	2	-
Fe^+2^ (mg/L)	1.3	1.3	1.5	1.3	0.4	0.4	0.6	0.3	0.4	0.3	20
S (as S^2−)^ (mg/L)	0.095	0.123	0.106	0.081	3.27	3.4	3.8	3.33	3.4	-	1

*P* denotes ponds from each site, *ND* is not detected

### Temperature

According to the European Spa Association (ESPA), the water, as it emerges from the spring must have a temperature greater than 20 °C to be officially classified as thermal and have therapeutic properties. Therefore, all the water qualifies to be called as thermal having therapeutic properties. Temperatures exceeding 42 °C are categorized as hot springs, those ranging from 35 to 42 °C as warm springs, and between 25 and 34 °C as tepid springs [10]. Therefore, Gasa Tshachu Pond 1 is categorized as warm with 41.3 °C and other three ponds above 42 °C categorized as hot springs. In contrast, Koma Tshachu featured tepid to warm waters (32.2 °C to 39.8 °C) and Chubu Tshachu recorded the highest temperatures (45.3 °C to 46.4 °C) and categorized as hot springs.

### pH

According to [11] hot springs are categorized as acidic (pH < 3), mildly acidic (pH from 3 to 6), neutral (pH from 6 to 7.5), mildly alkaline (pH from 7.5 to 8.5) and alkaline (pH >8.5). Therefore, Koma and Chubu Tshachu show alkaline water (pH 9.8–10.4), exceeding the WHO limit of 6.5–8.5. Gasa Tshachu falls within WHO safe range (7.9–8.0), indicating moderate alkalinity.

### Electrical conductivity & total dissolved solids

Chubu Tshachu recorded the highest electrical conductivity (EC) values, ranging from 816 to 817 µS/cm, which remain well below the WHO guideline of 2500 µS/cm for drinking water. Koma Tshachu exhibited even lower EC values, ranging from 419 to 520 µS/cm. In contrast, Gasa Tshachu displayed a remarkably low EC of 11.2 µS/cm, which should result in correspondingly low total dissolved solids (TDS), as calculated using the formula TDS = EC × 0.65. This would typically indicate a low concentration of dissolved minerals. However, as presented in Table 3, Gasa hot spring showed the highest ion concentrations among the sampled springs.

To confirm the accuracy of this unexpected result, the measurements were validated through three independent tests, ruling out possible errors related to instrument calibration or sample collection procedures. A plausible explanation for this discrepancy is the presence of ion pairing and strong ion–ion and ion–solvent interactions. These interactions can significantly reduce ion mobility by forming clusters or complexes, which in turn lowers the EC despite high levels of total dissolved solids [12].

Additionally, the relatively high turbidity observed in Gasa Tshachu may suggest elevated levels of organic matter. Organic compounds can bind with free ions, forming complexes that reduce the number of free, mobile ions in solution, thereby diminishing the measured EC. Previous studies have also reported that magnesium ions can contribute to reduced electrical conductivity due to similar mechanisms [13].

### Turbidity

WHO permissible limit is 0.5 NTU for drinking water (not strictly applicable to therapeutic soaking). Gasa shows high turbidity (7.9–10.9 NTU) and it could be due to suspended minerals or organic matter. Koma and Chubu is mostly within or near WHO limit (0.0–1.2 NTU).

### Total hardness (TH)

Chubu hot spring water is hard (41 mg/L CaCO₃) rich in calcium/magnesium ions, known for muscle relaxation. Koma varies from soft to moderately hard (11.3–33.3 mg/L). Gasa hot spring has low hardness.

Some individuals believe that drinking thermal water can alleviate gastroenterological ailments. Moreover, the water is considered sacred therefore some just sips and others drink sometimes. This study highlights that sodium levels (1695–1938 mg/L) and potassium levels (199–269 mg/L**)** in Gasa Tshachu far exceed the WHO drinking water standards (200 mg/L for Na⁺, 100 mg/L for K⁺), indicating it is not suitable for consumption. Other trace elements such as copper, iron and manganese are found high in all. Heavy metal were not tested however Gasa is reported to have above permissible limit of arsenic as per the study by Ministry of health. Therefore, drinking is not recommended.

Although the major ion concentration is sodium and chloride in all the ponds of the three hot springs, Gasa Tshachu was dominated by high concentrations of sodium, chloride, and bicarbonate, Chubu Tshachu had moderate sodium and chloride but higher sulfur (3.33–3.40 mg/L), sulphate and calcium which could be the reasons for different health benefits as perceived by users. Koma Tshachu showed higher levels of magnesium, carbonate, and sulfur. According to the European Spas Association, healing waters should ideally contain certain minimum levels of minerals for therapeutic use: calcium at 500 mg/L, magnesium at 150 mg/L, sulphate at 1200 mg/L, and bicarbonate at 1300 mg/L. However, the hot springs studied have mineral concentrations that are lower than these recommended values. Therefore, the observed therapeutic benefits of the hot springs suggest that healing effects arise from a combination of factors such as osmotic water pressure, temperature, mineral composition, and individual physiological responses.

A comparison between the physiochemical and mineral ion content of the hot springs in the Himalayan region is shown in Table 4. This is done to identify trends or anomalies that may indicate unique geological features or processes that shaped the hot spring’s healing properties. Most of the hot springs in the region are similar in their physiochemical properties, moderately alkaline in nature.

**Table 4:** Physicochemical properties of hot springs in the Himalayan region and this study

Parameters	Gandaki, Nepal [14]	Chutrun, Pakistan [15]	Sikkim, India [16]	Tibet, China [17]	This study
Temp (^O^C)	31.6–64.3	40.0–42.0	45.0–70.0	28.4–87	32.2–46.4
pH	7.30–8.80	7.21–7.80	7.40-8.0	6.68–9.78	7.9–10.4
EC (µs/cm)	206-16270	-	112–608	3.13–68,500	11.2–817
TDS (mg/L)	115–6637	300–310	238–1180	163–2021	7.28–531
Na^+^ (mg/L)	7.90–370	12.0–18.0	-	69.7–789	180–1937
Ca^2+^ (mg/L)	8.00-364	80.0–82.0	7.00–61.0	1.4-150.5	0-35.5
K^+^ (mg/L)	2.80–137	3.80–4.10	-	-	0.65–294
Mg^2+^ (mg/L)	1.90–121	20.0	2.00–25.0	0-53.5	0–33
Cl^−^ (mg/L)	1.90–121	9.60–12.0	12.0-109	0.7–63.5	85.3–288
NO_3_^−^ (mg/L)	0.00-2.60	-	0.050–3.50		2.4–34.
S^2−^(mg/L)	-	-	-	-	0.08–3.80
SO_4_^2−^ (mg/L)	0.000-24.9	80.0–85.0	3.40–21.2	2-120.9	2–24
HCO3^−^ (mg/L)	61.33–490	260–282	-	69.5-2205.8	1.6–330

### Hydrochemical facies classification

**Fig. 2:**
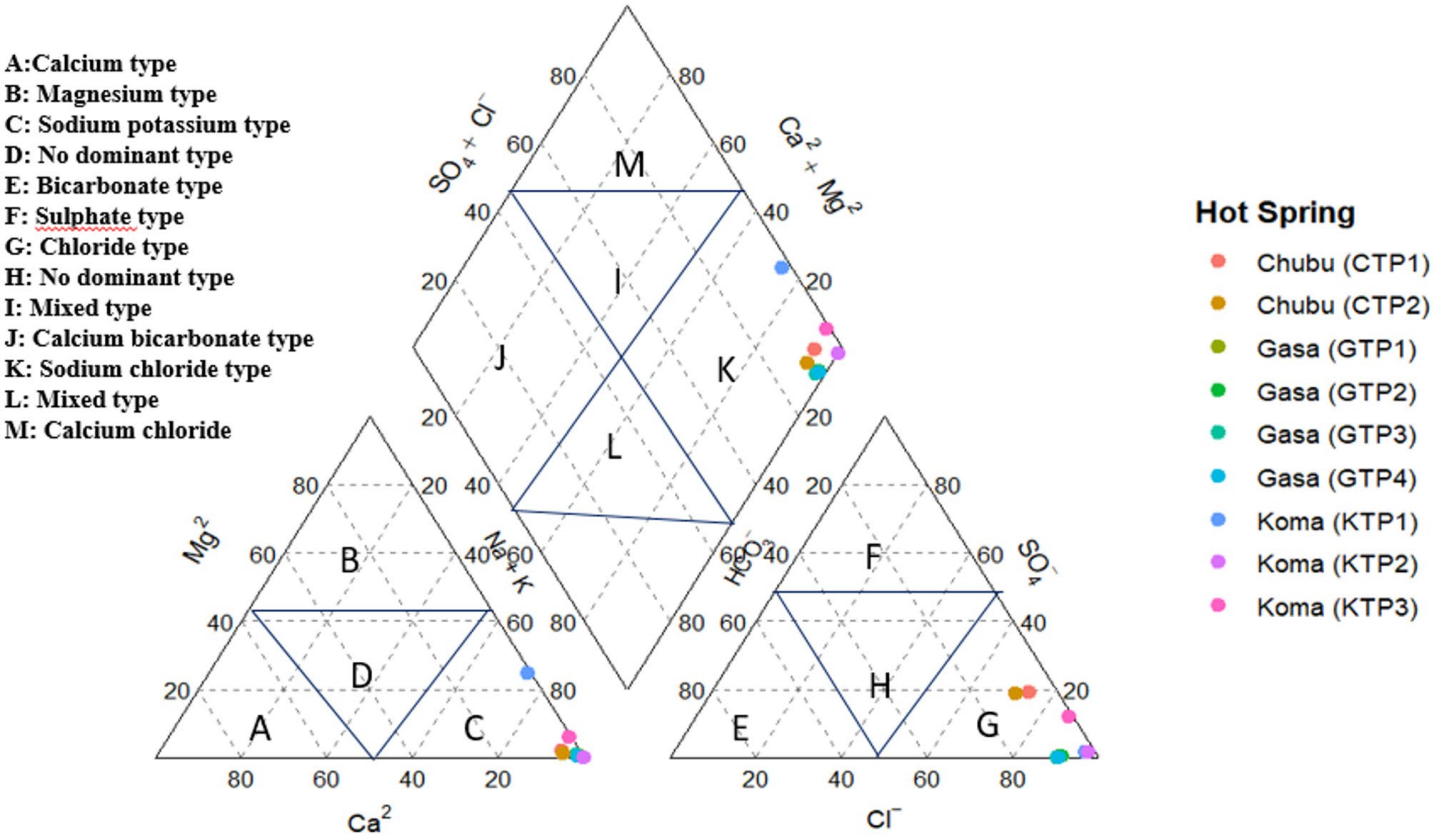
Piper diagram, showing hydrochemical facies of water (mEq/L%)

The hydrochemical composition of hot spring waters from Gasa, Koma and Chubu was classified using a Piper diagram (Fig. 2.). All samples plot predominantly in the sodium-chloride (Na⁺–Cl⁻) field (Zone K) in the central diamond, indicating a consistent hydrochemical facies across the study area. In the cation triangle, the waters are strongly dominated by sodium and potassium (Na⁺ + K⁺), while in the anion triangle, chloride (Cl⁻) is the major anion. The homogenous classification of water as sodium-chloride type is aligned with perceived balneological benefits, particularly for musculoskeletal (arthritis, rheumatism, swelling in joints, back pain) and dermatologic **(**skin diseases and itching disorders, pustules on face and body, facial beauty), conditions across all the hot springs.

**Fig. 3:**
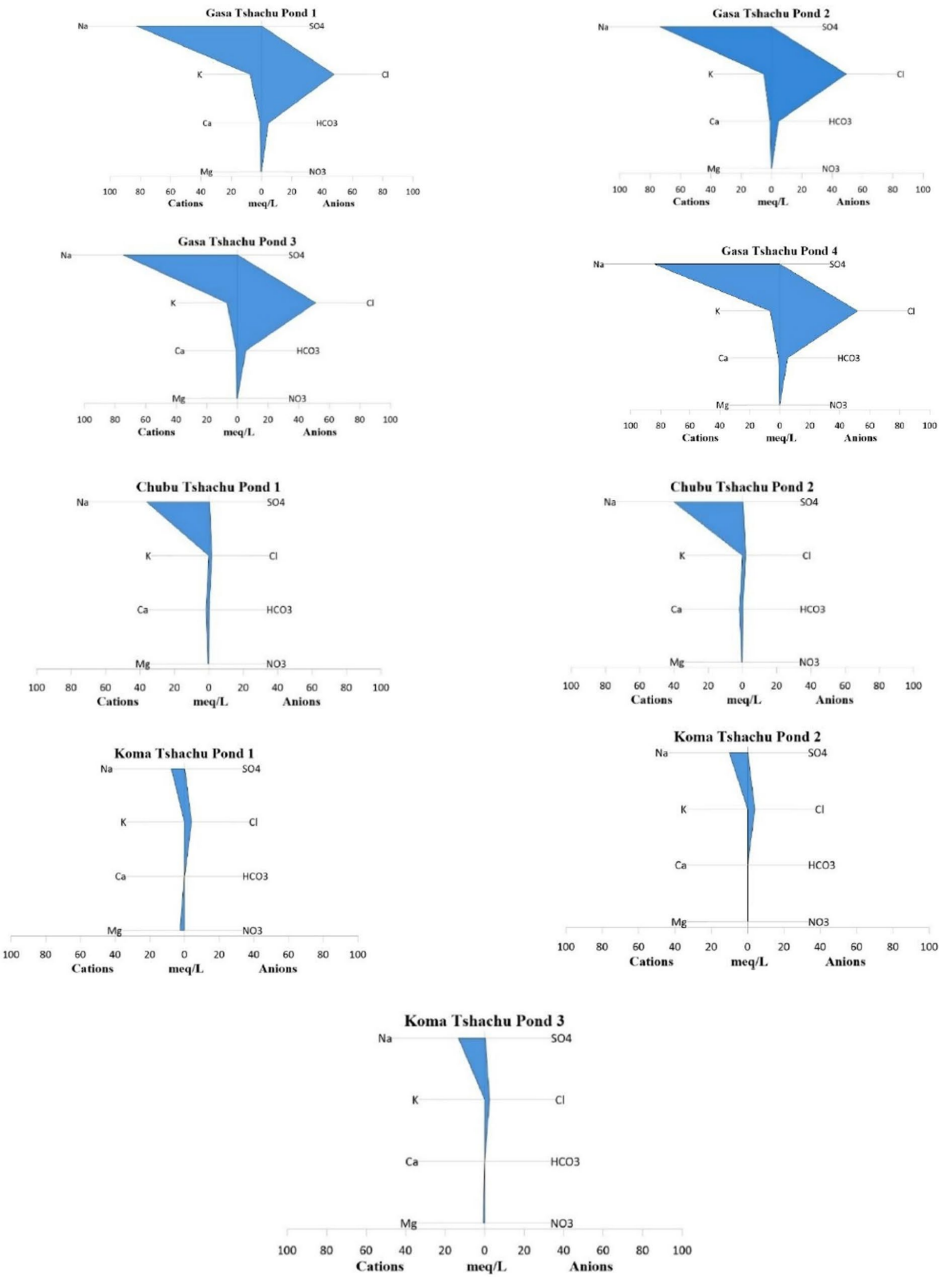
Stiff diagrams showing ion concentrations in milliequivalents per liter

A Stiff diagram (Fig. 3.) was used which shows the relative concentrations of major cations and anions in a single water sample as a polygonal shape, which was then compared across ponds of different hot springs to identify similarities or differences in water chemistry. In terms of similarity all the hot spring ponds have major sodium-chloride (Na⁺–Cl⁻) type ions, indicating a common hydro-geochemical signature dominated by these ions. Gasa hot springs have the maximum ion concentration of sodium and potassium, followed by Chubu and Koma. Similarly, chloride and bicarbonate content were also highest in Gasa.

### Relation between the therapeutic claims and the physicochemical properties of water

A Spearman’s rank-order correlation was conducted to explore the relationship between the physicochemical properties of thermal water and the perceived therapeutic effects across five health domains: musculoskeletal, dermal, respiratory, gastrointestinal, and cardiovascular. The results are presented in Table 5. The analysis showed that cations particularly calcium (Ca²⁺), magnesium (Mg²⁺), sodium (Na⁺), and potassium (K⁺) tended to have positive correlations with perceived improvements in musculoskeletal and dermal conditions.

**Table 5:** Spearman correlation coefficient (rho) between the perceived health benefits and the physicochemical properties of thermal water

	Water Temp	pH	Na^+^	K^+^	Ca^+2^	Mg^+2^	SO_4_^_^	Cl^−^	HCO_3_^−^	NO_3_^−^
Musculoskeletal	0.411	−0.348	0.411	0.418	0.548	0.55	0.207	0.137	0.481	0.07
Respiratory	−0.621	0.532	−0.434	−0.523	− 0.727*	0.427	−0.447	0.142	−0.125	0.05
Gastrointestinal	0	0.661	− 0.693*	−0.617	0.087	0.13	0.349	− 0.693*	− 0.870**	−0.178
Dermal	0.026	−0.523	0.448	0.456	−0.105	− 0.873**	−0.483	0.501	0.313	0.23
Cardiovascular	0.082	0.005	−0.137	−0.065	0.11	−0.623	0.41	−0.411	−0.417	−0.173

*Correlation significant at 0.05 level; **Correlation significant at the 0.01 level

These minerals are known to be beneficial in balneotherapy, particularly in rheumatic and musculoskeletal disorders. Calcium and magnesium can reduce muscle stiffness and improve joint function by facilitating neuromuscular transmission and acting as anti-inflammatory agents. Potassium can be used therapeutically to reduce joint pain and inflammation in rheumatoid arthritis [18]. Sodium is indispensable for muscle contraction and nerve signaling. In skin, it primarily supports hydration and barrier integrity. However, while the correlation suggests a potential link between mineral content and health benefits, it is important to acknowledge that other factors such as water temperature, pH, electrical conductivity, and the broader psychosocial environment may also significantly contribute to the perceived well-being of hot spring users.

All other correlations, such as that observed with bicarbonate which is traditionally recognized for its role in neutralizing stomach acid are challenging to interpret. This ambiguity may be attributed to the limited sample size, comprising only nine ponds and 36 participants, which could have affected the statistical power and reliability of the findings.

## Discussion

In Bhutan, hot springs are often associated with deities and spiritual beliefs, therefore are sacred sites and bestows blessings upon those who visit the hot springs. Therefore, it serves as a sanctuary for mental well-being and physical recovery through the therapeutic properties of the hot springs. Similarly in Japan, bathing in hot springs is found to have a positive effect on mental health [19].

The ethno-pharmacological properties of three prominent hot springs exhibited notable similarities in their therapeutic applications which could be explained by the similarities in the physiochemical properties. The water type found is sodium chloride in all the three hot springs which is similar with the results from the Himalayan regions such as Sikkim; Gandaki province, Nepal [14], Chutrun, Pakistan [15] and Tibet, China [17]. Moreover, according to [20], the physicochemical analyses of hot springs in Himalayan geothermal belt indicated the water mainly as sodium chloride (Na-Cl), Sodium bicarbonate (Na-HCO_3)_, predominantly sulphate chloride (SO_4_-Cl) and mixed type.

The pH of the water was alkaline comparable to other studies [14, 16], further indicating the carbonate dominated underlying lithology. Sulfur is a significant component found in hot springs, and as one approaches the Chubu hot spring, the characteristic odour of sulfur becomes noticeable. The Chubu and Koma Tshachu ponds exhibited similar concentrations ranging from 3.27 to 3.80 mg/L, whereas Gasa recorded a level of less than 1 mg/L. Moreover, Chubu pond 2 had the highest concentration of sulphate at 24 mg/L.

The healing properties of the hot springs are known to be the result of a combination of factors, with mechanical (hydrostatic pressure), thermal and chemical effects. The increased buoyancy and hydrostatic pressure experienced when immersing in thermal water leads to several physiological changes, including heightened urine production (diuresis), increased sodium excretion (natriuresis), and enhanced cardiac output [21]. This could be one the reasons for the blood pressure decrease as explained by respondents suffering hypertension.

Moreover, the thermal heat increases the secretion of stress hormone such as cortisol which plays a significant role in metabolism, immune response regulation, and blood pressure maintenance. Heat stimuli also release β-endorphin known for its potent analgesic properties [21]. Clinical studies have shown that prolonged bathing in hot spring in summer is not recommended due to significantly increased cardiac stress and a prominent drop of blood pressure should be concern especially for elderly in winter [22].

On average, Bhutanese dedicate around seven hours daily soaking in hot springs, however their duration of the stay ranges from one day to a week or more depending on the ailments. Studies have shown that soaking in hot spring for three and more days have significant therapeutic effects on patients with musculoskeletal disorders, including rheumatoid arthritis [23]. A three week long rehabilitation clinical studies (30 min each day for 5 days a week) reduced psoriasis inflammation [24]. According to [22] ideal period for hot spring soaking is 10 days with soaking time dependent on each person’s body adaptability and water temperature because if the temperature and the mineral concentration is high there will be initial aggravation of the symptom after taking a bath for one to two days which is actually the signal that the body is responding. Therefore, to get the optimum benefit, soaking for 10 days is recommended instead of soaking long hours in a day or two. Conversely, intermittent balneotherapy i.e. once weekly treatment for six weeks has been found to be effective in the treatment of knee osteoarthritis [25]. Therefore, this requires further research.

Saline thermal mineral waters with high sodium and chloride are found to be good for treating various musculoskeletal disorders, including joint conditions such as osteoarthritis (OA), rheumatoid arthritis, psoriatic arthritis, gout, spondylitis, and issues affecting the spine, including back and neck pain [26]. This could be the reason for most visitor stating the musculoskeletal benefits in all hotspring.

Further, hydrogen sulfide (H_2_S), the active component in sulfurous mineral waters, is gaining scientific interest for its therapeutic potential. As a gas, H_2_S can be absorbed through various routes, allowing it to reach internal organs. Its main functions include vasodilation (widening of blood vessels) and promoting new blood vessel growth [27] therefore helps in controlling high blood pressure (BP). However, the contraindication is that those patients with high blood pressure or heart problems should not be soaking in water for long [28]. One respondent in Chubu reported using shorter intervals to manage her hypertension, underscoring the importance of individualized practices. Sulfur promotes keratolysis, aiding in the shedding of the outer skin layer, which helps treat chronic venous leg ulcers and eczema. Additionally, sulfur-rich water is found to reduce inflammation on mucous membranes, making them beneficial for treating respiratory conditions such as sinusitis, bronchitis, and asthma [29]. Other therapeutic indications of sulphur are treating urinary tract infections [26] and was stated by respondents for the upper pool in Chubu Tshachu. Sulfur in the hot springs is also believed to smoothen and moisturize the skin, a benefit that was reported by few young respondents. This skin benefit could be triggered by sulfur penetrating the skin, causing particular dilation of blood vessels with higher temperatures of the water [30]. Sulphur is also found to have bactericidal and fungicidal properties [31].

Hot spring water rich in sodium has been found to enhance the effectiveness and tolerability of acne treatments, leading to improved skin hydration and a greater sense of skin suppleness [32]. This could be the reason that some respondents in Gasa hot spring stated their skin felt smooth after soaking. Studies have reported that magnesium-rich Dead Sea salt solutions enhance the protective role of skin, boost hydration, and decrease inflammation in atopic dermatitis [33]. Calcium bicarbonate water is known for its anti-inflammatory, soothing, and healing effects and is used to treat acne and burns [29] which could be one of the reasons for facial beauty expressed by young adults.

According to a few respondents, Chubu hot spring (CTP2, lower one) is believed to help with diabetes-related symptoms which could be supported by clinical trial results on mice which indicated that water from Süreyya hot spring in Turkey was successful in the treatment of diabetes mellitus [34]. However, it should be noted that such claims are anecdotal and needs to be backed with clinical verification.

Despite many benefits, general contraindications for balneotherapy should also be considered. These contraindications include severe psychiatric conditions, acute alcoholic states, epilepsy, severe varicose veins, open wounds and if hypersensitive to mineral baths [35], acute illnesses, particularly those with fever, active tuberculosis, bleeding disorders, advanced anemia infectious diseases, malignant tumors, severe heart problems, respiratory or renal failure, or during the first and last trimesters of pregnancy [28] Therefore, precautions are necessary for elderly or those with specific health conditions.

## Conclusion

The study found that the sacred nature of hot spring sites, often associated with deities, plays an important role in shaping community beliefs and healing practices. Users often travel long distances and remain for extended periods, indicating the strong cultural and therapeutic importance attached to these waters. Sixteen healing perceptions were documented and categorized into five group, musculoskeletal, dermal, gastrointestinal, respiratory and cardiovascular. Physicochemical analysis showed that all ponds of hot springs of Gasa, Koma, and Chubu are characterized by sodium-chloride (Na⁺–Cl⁻) water type with varying levels of bicarbonate, sulfate, calcium, and magnesium. Moderate positive correlations between perceived healing and physiochemical properties were found for Na⁺, K⁺, Ca^+2^ and Mg^+2^ for musculoskeletal and dermal. While most parameters were within WHO guidelines, some ion concentrations (e.g., Na⁺, K⁺, Mg) exceeded permissible limits for drinking, underscoring that the waters are more suited for balneotherapy. Chubu and Koma hot springs, with higher sulfur and sulfate levels, were associated with improved wound healing, respiratory relief, and skin health, supported by both respondent narratives and scientific literature. The higher alkalinity of Koma may also contribute to accelerated healing effects, particularly in fractures and gastric issues, as reported by users. However, it requires further studies supported by clinical trials.

### Recommendations

- Conduct a nationwide survey and scientific assessment of all hot springs in Bhutan to establish a baseline for water quality, ion concentrations, and therapeutic categories.
- Develop national standards and classification systems for hot spring waters based on their mineral content, pH, temperature, and potential health benefits.
- Conduct clinical trials and health outcome studies.
- Promote Bhutan as a destination for geohealth tourism, where visitors experience both healing and cultural immersion in a natural, sacred setting.

### Limitations of the study

The sample size was small for both sociocultural and physciochemcial analysis. Data collection period in Koma and Chubu did not coincide with the usual bathing season for large sample size. Furthermore, although this study established a connection between balneotherapy and mental well-being, the inclusion of a supplementary survey to strengthen these findings could not be undertaken due to time limitations.

The Total Suspended Solids (TSS) was not directly measured in this study; instead, it was estimated using electrical conductivity (EC) and a constant conversion factor of 0.65. This indirect estimation introduces a limitation, as it may not accurately reflect actual TSS concentrations under varying water chemistry conditions as was found in this study. There was overlap in perceived health benefits across all hot springs, which limited the ability to associate specific effects with individual water properties although the overlap could be because the major ions were found to be sodium and chloride. The study could not establish a causal link between mineral composition and therapeutic effects, as it was based on self-reported perceptions and lacked experimental validation. Nevertheless, this study provides a foundational study and results are generalized relating to the existing literature on the balneotherapy as clinical trials were beyond the scope of this study.

## Data Availability

The authors declare that the data supporting the findings of this study are available within the paper. Should any raw data files be needed, they are available from the corresponding author on reasonable request.
